# Different Aspects of Fatigue Experienced by Patients Receiving Maintenance Dialysis in Hemodialysis Units

**DOI:** 10.5812/numonthly.11667

**Published:** 2013-08-20

**Authors:** Vajihe Biniaz, Ali Tayybi, Eghlim Nemati, Mehdi Sadeghi Shermeh, Abbas Ebadi

**Affiliations:** 1 Nephrology and Urology Department, Baqiyatallah University of Medical Sciences, Tehran, IR Iran; 2 Department of Medical Surgical Nursing, Baqiyatallah University of Medical Sciences, Tehran, IR Iran

**Keywords:** Renal Insufficiency, Chronic, Kidney Failure, Chronic, Fatigue, Renal Dialysis

## Abstract

**Background:**

Fatigue, a common symptom reported by patients receiving dialysis, is a multidimensional and subjective experience which is readily understood by individuals but difficult to measure.

**Objectives:**

This study was performed to identify the prevalence of differential aspects of fatigue among patients receiving maintenance dialysis.

**Patients and Methods:**

The cross-sectional study was conducted in two hemodialysis wards in Tehran with a sample of 163 participants. In this study, the multidimensional fatigue inventory was used to determine the level of fatigue. Demographic data were also collected with self-report survey. To analyze data with SPSS statistical software, test Chi square, T-test, and ANOVA were used. P- Value less than 0.05 was considered significant.

**Results:**

All the patients experienced degrees of fatigue and 50 (30.7%) of the participants experienced a high level of fatigue. Fatigue scores arrangement was founded for physical fatigue followed by reduced activity and general fatigue. Lower levels of fatigue were reported for mental fatigue and reduced motivation. There was no diversity in this study in the levels of fatigue in respects of gender and marital status and employment status. Participants with diabetic nephropathy were the most fatigued.

**Conclusions:**

People with chronic kidney disease regardless of their age, gender, state of health, and duration of hemodialysis experience high levels of fatigue; it is particularly important for health providers to understand this level of fatigue which affects the daily life of patients.

## 1. Background

End-stage renal disease (ESRD) is one of the most common chronic diseases (1) which its incidence and prevalence are on the rise (2). Although patients receiving dialysis now live longer, most of them experience symptoms that interfere with their ability to function according to their normal capacity and hampering the quality of life (3).

Fatigue is one of the most common (60-97%) complications which hemodialysis patients are faced with (4). Fatigue is difficult to be measured objectively, and is an unpleasant subjective symptom (5). Although the need to identify and assess this real problem, which its cause remains unknown for patients receiving dialysis, is vital to patient health and quality outcomes (4), it is viewed by health providers as something that cannot be altered and as a part of the disease process (6), frequently unrecognized, and therefore undertreated. This may explain why fatigue has received relatively little attention in the literature of health providers (7). Health providers are in an excellent and strategic position to assist patients to explore and invigorate their coping mechanisms (4), and also to make ready a home care scheme to enhance coping with the symptoms of fatigue at home (3).

Fatigue is a multidimensional concept which involves general, mental, and physical fatigue, reduced motivation, and reduced activity dimensions (6). Despite excessive number of patients receiving dialysis and transplant in Iran (exceeded 50,000 in 2011) (8), no examination has been sufficiently reported to discriminate between the experience of differential dimensions of fatigue in Iranian ESRD patients. Understanding the variability of experience among five dimensions of fatigue would be helpful to develop therapeutic interventions and fatigue management (9).

## 2. Objectives

This study was performed to identify the prevalence of differential aspects of fatigue among patients receiving maintenance dialysis.

## 3. Patients and Methods

This study was directed from October 2012 to January 2013 by using a cross-sectional design on 163 patients receiving maintenance dialysis in two hemodialysis units in Baqiyatallah and Chamran hospitals of Tehran, Iran. The sampling frame included all ESRD patients who were older than 18 years, regular recourse for maintenance hemodialysis two or three sessions per week, and receiving dialysis for ≥ 3 months.

The research instrument consisted of two parts; a demographic questionnaire and the multidimensional fatigue inventory MFI-20 to measure fatigue. A demographic questionnaire was developed by the researcher assessment of age, gender, marital and education status, employment status, income, weight, nephropathy cause and duration of receiving dialysis.

The MFI-20 questionnaire is a 20-item (5 subscales and every subscale consists of four items) self-report instrument which is planned to measure fatigue. It includes five dimensions: general, physical and intellectual fatigue, reduced activity, and motivation. Each item can be rated between 1 and 5 and each subscale between 4 and 20. A higher score demonstrates a greater level of fatigue. The respondents had to compare each of the 20 declarations on how they had been feeling lately. 

The MFI-20 has been extensively used and achieved a fine internal constancy with an average α-coefficient between 0.65 and 0.89. The statistical associations (P < 0.001) between each of the subscales and the visual analogue scale indicated that convergent validity of the MFI-20 is good. An average Chronbach’s α coefficient of 0.89 and statistical correlations with daily activity scores, depression and anxiety, reduction in activity, and emotional distress demonstrated that internal consistency and construct validity of the MFI-20 are good as well (10).

The study participants were empowered to complete the questionnaire at home, at the hospital, away from the hospital environment and free from demands of the hemodialysis treatment which can be exhausting. 

Prior ethical approval was obtained from the institutional ethical committee at Baqiyatallah university of medical sciences, Tehran, Iran. A justification letter was sent to two hemodialysis units and permission obtained to collect data granted by these units. From all those who participated in the study verbal and written consents were obtained. Written consents were obtained after informing each participant about the study purposes, the “confidentiality” of their information, and the possibility to refuse the test procedure at any stage of it.

The data were analyzed by version 18 SPSS software. The χ2-test, T-test and ANOVA were used to determine the result of each variable on fatigue. The researcher decided to receive only a 5% error in rejecting of the full hypothesis at the 95% confidence interval. The significance level was put at 0.05.

## 4. Results

The demographic data of the participants showed that of the 163 respondents 100 (61.3%) were male and 63 (38.7%) female. The mean age of respondents was 61.39 years (SD 12.61) (range 24–88 years). Most of the respondents (54%) aged between 40-65. The mean age of males (61.4 years) was similar to that of females (61.3 years). Most participants (84%) were married, 12.9% and 3.1% were widowed and single, respectively. Most of the participants were retired (60%) with a mean age of 63 years. The mean age for unemployed and employed individuals were 62 and 47.5 years, and the average of hemodialysis duration was 37.6 months (SD 43.8; range 3–300 months) (more information exhibited in Table 1). 

All patients experienced fatigue, especially high fatigue scores were associated with reduced activity, physical fatigue and general fatigue from the MFI. The mean of fatigue was found for three of the five subscales; reduced activity (mean = 15.49, SD = 4.1), physical fatigue (mean = 15.2, SD = 3.8), and general fatigue (mean = 14.66, SD = 3.4). Lower levels of fatigue were reported for mental fatigue (mean = 9.7, SD = 3.9) and reduced motivation (mean = 10.3, SD = 3.6).

**Table 1.** Demographic Characteristics of the Respondents (n = 163)

Characteristic	Frequency, No. (%)
**Age, y**	
< 40	11 (6.7)
40–65	88 (54)
> 65	64 (39)
**Education**	
Primary	70 (42.9)
Under diploma	15 (9.2)
Diploma	46 (28.2)
University	32 (19.6)
**Weight**	
< 50	4 (2.5)
50-70	75 (46)
70-90	62 (38)
> 90	9 (5.5)
**Nephropathy cause**	
HTN	58 (35.6)
DM	23 (14.1)
Glomerulonephritis	6 (3.7)
HTN and DM	47 (28.8)
Others	29 (17.8)
**Income**	
Poor	31 (19)
Middle	105 (64.4)
Well	27 (16.6)
Total	163 (100)

Among the male respondents, 20 (12.1% of male patients) experienced a low level of fatigue, 50 (30.6%) experienced a middle level of fatigue; whereas, the other 30 (18.6%) experienced a high level of fatigue. 10 (6.2%) of female respondents experienced a low level of fatigue, 33 (20.2%) experienced a middle level of fatigue, and 20 (12.3%) experienced a high level of fatigue.

The mean of fatigue score in females and males were respectively 66.6 (SD 15.7) and 64.6 (SD 16), and no meaningful difference was shown in fatigue scores between them.

One-Way ANOVA showed a positive statistical association between age and some of the fatigue dimensions such as general fatigue, physical fatigue, reduced activity and motivation. As seen in Table 2, there was no significant correlation between age group (lower than 60, and older than 60) and total fatigue score. All participants irrespective of their age had experienced fatigue. 

**Table 2.** Association Between the Respondents' Age Group and Fatigue (n = 163)

Variable Age, y	Fatigue Level			P-value
	Low, No. (%)	Middle, No. (%)	High, No. (%)	
**< 60**	17 (10.4)	35 (21.5)	19 (11.7)	0.248
**> 60**	13 (8)	48 (29.4)	31 (19)	0.251

A significant difference was found between reduced activity and motivation dimensions and employment status. Thus employed patients had more levels of activity and motivation than unemployed, retired or housekeeper patients. But an ANOVA between groups showed no statistical correlation amidst fatigue dimensions and marriage status.

The mean duration of hemodialysis was 3.1 years. One-Way ANOVA revealed a significant positive association amidst dialysis vintage and some fatigue dimensions such as physical fatigue, reduced activity and reduced motivation dimensions. Pearson correlation also found a weak correlation between length of hemodialysis and total scores of fatigue, however, this was not statistically significant (P = 0.07).

The Hb mean in patients who experienced low, middle and high levels of fatigue were 11.33, 10.78, and 11.21 respectively. Although there was a contrary association between fatigue dimensions scores and Hb values, no statistical correlation between total fatigue scores and anemia was established. 

Comparing the overall fatigue mean scores with the cause of renal failure it can be concluded that the highest mean was recorded for patients with diabetic nephropathy (mean = 69.78, SD 14.65). Those ESRD due to diabetes and hypertension (mean = 68.38, SD 14.08) and HTN (mean = 66.39, SD 15.84) had the lowest mean fatigue scores.

## 5. Discussion

This study indicated that people with ESRD are faced with considerable fatigue, which would occur irrespective of patients' age, gender, state of health, and duration of hemodialysis. Our study results also demonstrated that fatigue is one of the most common hemodialysis side-effects which is supported by earlier studies (11).

Overall, the prevalence of fatigue was 65.4%, and 50 (30.7%) of the respondents experienced a high level of fatigue, 83 (50.9%) experienced a middle level, and 30 (18.4%) experienced a low level of fatigue. In similar studies, hemodialysis patients had a mean age of 61 years, 46.5 years, and 58.7 years (12) which is consistent with the present study results (61.3 years).

In O'Sullivan study the highest percentages of respondents were reported married (46%) and unemployed (65%) (5). In the present study most respondents were also married (84%), but retired (60%).

Results demonstrated that the highest fatigue scores were achieved for reduced activity followed by physical fatigue and general fatigue. In some studies, the arrangement of fatigue scores was found for physical fatigue followed by reduced activity and general fatigue (5). This indicates that physical functioning abilities may differ among hemodialysis patients. The reason of this finding may be related with confounding variables such as sufficiency of hemodialysis and the existence of sleep disturbances which may affect the fatigue experience. The physical activity restrictions in exhausted patients can influence self-care abilities, employment activities, and social roles. It can also impose additional costs on individuals, community, and the insurance services. The least fatigue level experienced was mental fatigue and reduced motivation that showed motivation and concentration abilities retained flawless.

The nonconformity of fatigue scores between male and female is supported by some similar studies (13). Overall females reported more fatigue compared to males, but this difference was not significant (66.6 via 64.6), unlike Brunier and O'Sullivan studies who reported higher scores of fatigue in men or women. However female participants articulated their feelings and less motivated when fatigued, these gender differences may be associated with biological and cultural factors.

Similarly from some studies such as O'Sullivan we found a significant association amidst dialysis vintage and some fatigue dimensions; however, there was not a significant association between total fatigue scores and duration of hemodialysis. This finding was also supported by some other studies, for example McCann (13) and Karakan (14). It may be related to coping behavior alterations in hemodialysis patients and their accustoming to routine dialysis.

The age examination in participants demonstrated a statistical positive association between age and some of the fatigue dimensions; however, all of the participants who were < 60 years old and or were > 60 years inclined to get fatigued. The participants who were in the working group, < 60 years old, experienced fatigue because of labor of coping with job tasks. These results were similar to those of O'Sullivan’s. Unruh (15) who reported that older patients in his study experienced a greater level of fatigue. Although lack of information about fatigue handling systems could have impacted on it, the effects of physiological alterations which occur with age were also influential.

Fatigue is a symptom of anemia (16), but the present study did not show any correlation between hemoglobin laboratory values and fatigue. Also, Bonner (6), Karakan (14) and Letchmi (3) did not report any association between higher levels of fatigue and anemia. Apparently, there is a complex association between fatigue, anemia and symptom presentation, which requires further clarification (17).

The sampling frame was one of the limitations in our study. The cross-sectional plan used restricted the ability to generalize findings and to detect associations between fatigue levels and situational factors.

Conclusion: people with chronic kidney disease experience high levels of fatigue regardless of their age, gender, state of health and duration of hemodialysis. The experienced fatigue in reduced activity, physical fatigue and general fatigue aspects was more of other aspects in these patients. Understanding the level of fatigue in these patients and its effects on their daily living would help to qualify life improvements modifications.

## References

[1] Castner D (2010). Understanding the stages of chronic kidney disease.. Nursing..

[2] Horigan AE (2012). Fatigue in hemodialysis patients: a review of current knowledge.. J Pain Symptom Manage..

[3] Letchmi S, Das S, Halim H, Zakariah FA, Hassan H, Mat S (2011). Fatigue experienced by patients receiving maintenance dialysis in hemodialysis units.. Nurs Health Sci..

[4] Horigan A, Rocchiccioli J, Trimm D (2012). Dialysis and fatigue: implications for nurses--a case study analysis.. Medsurg Nurs..

[5] O'Sullivan D, McCarthy G (2007). An exploration of the relationship between fatigue and physical functioning in patients with end stage renal disease receiving haemodialysis.. J Clin Nurs..

[6] Bonner A, Wellard S, Caltabiano M (2008). Levels of fatigue in people with ESRD living in far North Queensland.. J Clin Nurs..

[7] Bonner A, Wellard S, Caltabiano M (2010). The impact of fatigue on daily activity in people with chronic kidney disease.. J Clin Nurs..

[8] Aghazamani M (2011). [20% increase to renal patients yearly].. Salamat J..

[9] Lee BO, Lin CC, Chaboyer W, Chiang CL, Hung CC (2007). The fatigue experience of haemodialysis patients in Taiwan.. J Clin Nurs..

[10] Smets EM, Garssen B, Bonke B, De Haes JC (1995). The Multidimensional Fatigue Inventory (MFI) psychometric qualities of an instrument to assess fatigue.. J Psychosom Res..

[11] Savari S, Tayybi A (2013.). [Effect of receiving intravenous vitamin B12 on fatigue in patients undergoing hemodialysis].. Iran J Crit Care Nurs..

[12] Cleary J, Drennan J (2005). Quality of life of patients on haemodialysis for end-stage renal disease.. J Adv Nurs..

[13] McCann K, Boore JR (2000). Fatigue in persons with renal failure who require maintenance haemodialysis.. J Adv Nurs..

[14] Karakan S, Sezer S, Ozdemir FN (2011). Factors related to fatigue and subgroups of fatigue in patients with end-stage renal disease.. Clin Nephrol..

[15] Unruh M (2008). Sleep disorders in chronic kidney disease.. Prim Psych..

[16] Penninx BW, Pahor M, Cesari M, Corsi AM, Woodman RC, Bandinelli S (2004). Anemia is associated with disability and decreased physical performance and muscle strength in the elderly.. J Am Geriatr Soc..

[17] Ossareh S, Roozbeh J, Krishnan M, Liakopoulos V, Bargman JM, Oreopoulos DG (2003). Fatigue in chronic peritoneal dialysis patients.. Int Urol Nephrol..

